# Supplementary material for: Cost-effectiveness uncertainty may bias the decision of coal power transitions in China
Source: Nat Commun. 2024 Mar 13;15:2272. doi: 10.1038/s41467-024-46549-5 (PMC11639715; doi:10.1038/s41467-024-46549-5)
Supplement: Supplementary file 1 — Supplementary Information [file 41467_2024_46549_MOESM1_ESM.pdf]

Supplementary Information of

Cost-effectiveness uncertainty may bias the decision of coal power transitions in China

Xizhe Yan[1], Dan Tong[1,*], Yixuan Zheng[2], Yang Liu[1], Shaoqing Chen[3,4], Xinying Qin[1], Chuchu Chen[2,5], Ruochong Xu[1], Jing Cheng[1], Qinren Shi[5], Dongsheng Zheng[1], Kebin He[5,6], Qiang Zhang[1], Yu Lei[2,*]

[1]Department of Earth System Science, Ministry of Education Key Laboratory for Earth System Modeling, Institute for Global Change Studies, Tsinghua University, Beijing, People's Republic of China
[2]State Environmental Protection Key Laboratory of Environmental Pollution and Greenhouse Gases Co-control, Chinese Academy of Environmental Planning, Beijing, People's Republic of China
[3]School of Environmental Science and Engineering, Sun Yat-sen University, Guangzhou, People's Republic of China
[4]Guangdong Provincial Key Laboratory of Environmental Pollution Control and Remediation Technology, Sun Yat-sen University, Guangzhou, People's Republic of China
[5]State Key Joint Laboratory of Environment Simulation and Pollution Control, School of Environment, Tsinghua University, Beijing, People's Republic of China
[6]Institute for Carbon Neutrality, Tsinghua University, Beijing, People's Republic of China

*corresponding authors: dantong@tsinghua.edu.cn; leiyu@caep.org.cn

Supplementary Text 1. The potential impact of new-built capacity

Supplementary Fig. 9 shows the trends in coal power generation, deaths, and CO_2 emissions attributed to new-built capacity for each strategy. For all strategies, the overwhelming magnitude of new-built coal power capacity is important in shaping the future power fleet, with distinct roles played by new-built capacity in targeted strategies compared to the Historical strategy. Across all targeted strategies, new-built units, accounting for 65-67% of total installed capacity, would contribute to over 3700 TWh coal power generation and 2.5 Gt CO_2 emissions in 2030. Meanwhile, under the Historical strategy, new-built units would only account for 45% of total installed capacity with 1.7 Gt CO_2 emissions due to the slower phaseout pace.

New-built units are expected to dominate coal power fleet after 2040, as the majority of existing units will have been phased out in targeted strategy. For example, nearly 90% of coal power generation is generated by new-built units in all targeted strategies by 2045, contrasting with a 48% dependence on new-built units within Historical strategies. The slower phaseout pace in Historical strategy has prompted increasing CCUS retrofitting in existing units, enabling continuous operation beyond their 40-year lifetime.

Supplementary Fig. 8 shows the contribution of phaseout of existing units and construction of new-built units to the uncertainty of annual net benefits within each strategy. The construction of new units contributes to a certain level of uncertainty in net benefits of phaseout decision. For Health strategy, 40%-50% of the total benefit uncertainty of phaseout benefit could be attributed to the new-built power units before 2040. The contribution of new capacity keeps increasing to around 80% in 2050. This is mainly due to the uncertain selection of sites for new-built capacity has a significant impact on power-related health risks' uncertainty. Thus, choosing suitable site for constructing new coal-fired power plants can effectively mitigate the health risks associated with coal power and alleviate the risks brought about by uncertainty.

Supplementary Text 2. Baseline test for energy efficiency of new-built capacity

Assessing the potential impact of energy efficiency of new-built capacity is necessary in our study, enabling an analysis of how energy efficiency improvement would affect the benefit of targeted phaseout strategies. On one hand, new-built units will gradually take the lead after 2040, assuming average lifetime of 30-40 years for all existing coal power units. On the other hand, proposed coal power projects are subject to more stringent clean requirements since 14th Five-Year Plan[1]. Quantitative requirement of ultra-supercritical units with coal consumption rate lower than 270 gce/kWh are recommended, which is more advanced than the energy efficiency of current coal power fleet in 2018 (i.e., 307.6 gce/kWh).

Supplementary Fig. 10 shows that the effect of decarbonization is more obvious across all targeted strategies, when taking the advancement in combustion technology into consideration, by using a Historical strategy where new-built capacities would operate at the current energy efficiency level as benchmark. Carbon strategy, which is designed for decarbonization, can reduce over 7 Gt emissions during 2018-2060. This reduction is equivalent to the emissions produced by coal power over a two-year period.

In this baseline test, the net benefits of all targeted strategies would remarkably increase. For example, the cumulative net benefit of Carbon strategy increased from -378 (CI, -527~-231) to 215 (CI, 65~362) billion RMB if the energy efficiency of new-built capacity in Historical is set to 307.6 gce/kWh. This is due to replacing backward generating units with other inefficient units would not result in a swifter pace of efficiency improvement. It might restrict the potential benefits associated with decarbonization and mortality reduction within Historical strategy of baseline test. To prevent falling into a new cycle of technological lock-in and mitigate the risk of overall negative benefit, it is imperative to replace outdated generating units with efficient ones in a cost-effective manner.

Supplementary Text 3. Sensitivity test of future phaseout rate

The decision to phaseout a coal-fired power unit is usually driven by economic factors of operating costs, replacement costs, and revenues[2]. Globally, coal plants have retired at an average lifetime of over 45 years, thus, a lifetime of 40 years for power plant is widely adopted within the research community[3-5]. However, it is worth noting that China has actively mandated the replacement of outdated generating capacity with more advanced and younger power plants since the 11th five-year plan, resulting in a shorter operational lifetime[6]. A sensitivity test is conducted to evaluate how changes in phaseout rate would affect the cost-effectiveness of the phaseout decision.

Supplementary Fig. 12 shows the comparisons of cumulative cost-benefits and uncertainties for each strategy between the original scenario (setting a lifetime of 40 years for Historical strategy and a faster retirement rate of 40% in 2030 and 100% in 2050 for other strategies) and a sensitivity test with faster phaseout rate (setting a lifetime of 30 years for Historical strategy and a faster retirement rate of, 40% in 2025 and 100% in 2040 for other strategies). The risk of cumulative negative outcomes substantially increases. For example, the probability of Age-to-Capacity strategy experiencing cumulative negative outcomes rises to over 80%. This is because an accelerated phaseout rate for all strategies would diminish the relative cumulative benefits of health and decarbonization of targeted strategies compared to the Historical strategy, by a more rapid introduction of new-built capacity. Supplementary Fig. 25 result shows Age-to-Capacity strategy would favor as the optimal strategy in more preferences for its advantage in asset stranding avoidance. This is due to the relative gap in normalized asset stranding is further amplified, within a faster phaseout pace. It is essential to reduce the policy implementation disruptions and minimize uncertainties, especially in light of the heightened risk of negative benefits.

Supplementary Text 4. Sensitivity test for annual utilization hours

In recent years, the annual utilization hours of coal power have been declining brought out from overcapacity[7]. It might continue to decrease in the future with the call for flexibility transformation. Given diverse trajectories, uncertainty exists in forecasting the operating hours of coal-fired power plants. Sensitivity test for annual utilization hours is added by removing the assumption of a year-by-year decrease in capacity factor.

Supplementary Fig. 14 shows that the net benefit for all targeted strategy would further amplify, if keep the original annual utilization hours. For example, the net benefit of Carbon strategy would increase to 95 (CI, -197~275) billion RMB. The probability to achieve a positive outcome is around 70%. This is because over 40% of generation are attributed to existing units with lower phaseout priorities and better performance in 2030, if their annual utilization hours are not forced to decrease. When it comes to preference-based phaseout decision, limited changes compared to the original scenario (Supplementary Fig. 26), because the gap of normalized benefit and cost remains stable in both scenarios.

Supplementary Text 5. Sensitivity test of CCUS priority

Supplementary Fig. 15 shows a suitable allocation of CCUS installation among new-built and existing units may increase the net benefit of phaseout policy. For example, the cumulative net benefits of Age-to-Capacity and BAU strategies increase to 451(CI, 292~606) and 541 (CI, 372~707) billion RMB, when allocating 50% of the CCUS demand to new-built units and assigning the remaining demand to existing units. This is because more than 250 GW of existing units with lower phaseout priorities, which might have better performance (e.g., equip with better emission control technology) than new-built units, are involved into CCUS retrofitting. Extending the operational lifespan of those advanced units can contribute to reducing both health risks or CO_2 emissions associated with coal power, which highlights the importance of strategically retrofitting advanced coal power units with CCUS. Hence, Age-to-Capacity, Health and Carbon strategies have the potential to confer a competitive edge in preference-based decision-making compared to BAU strategy (Supplementary Fig. 19).

Supplementary Text 6. Case study of Inner Mongolia

Coal power will be a crucial lever for Inner Mongolia to achieve clean air and climate mitigation. Benefiting from abundant coal and renewable resources, as well as developed external power transmission grids, Inner Mongolia consistently takes the lead in outbound electricity since 2013 and becomes a major energy base in northern China. With a relatively young and extensive coal power infrastructure (total capacity of 79.8 MW and averaged age of 9.3 in 2018), coal power generation in Inner Mongolia reached 388 TWh in 2018, ranking third nationally after Shandong and Jiangsu. The low-carbon and green transition of coal power holds importance in this region, due to the coal-dominated energy structure and high proportion of industrial electricity usage. Therefore, we choose Inner Mongolia as a case to assess the potential outcomes and its uncertainty of coal power phaseout policy.

Inner Mongolia might face a unignorable risk of achieving negative net benefits (Supplementary Fig. 18a). Taking BAU strategy as an example, the probability of achieving negative net benefits is 30%, much higher than the national average level. This is due to the huge asset stranding when implementing early phaseout of young power fleet in Inner Mongolia. Nevertheless, Health and Age-to-Capacity strategies are likely to ensure net positive net benefit in future turnover. Such targeted phaseout strategies can accelerate the replacement of small power plants with cleaner and larger ones, leading to an increasingly clustered distribution of coal power (Supplementary Fig. 18b). Despite the increasing coal power demand, the number of coal-fired power units in Age-to-Capacity have decreased from 378 to 284 during 2018-2030, with an averaged installed capacity per unit reaching 500 MW. Within Historical strategy, there would still be 207 units with an averaged installed capacity below 300 MW in 2030.

Supplementary Fig. 20 shows phaseout strategies selection tailored to different policy preference in Inner Mongolia. The prior phaseout practice (i.e., BAU strategy) might not be the optimal choice given various decision preferences (Supplementary Fig. 20a), which is different from the nationwide preference-base phaseout decision. An alternative and suitable phaseout strategy should be sought for the future decarbonization of coal power, according to the objectives set by policy-makers. Uncertainties may still bias the preference-specified phaseout decision to a certain degree in Inner Mongolia (Supplementary Fig. 20b-e). Therefore, it is necessary to thoroughly assess the emissions and operational conditions of all units and implement such a unit-by-unit phaseout plan in a finer level.

Supplementary Text 7. Sensitivity test of future coal power demand

Existing research offers diverse mitigation pathways for coal-fired power plants in China, some of which suggest generation of coal power will decrease to lower levels, driven by accelerated renewable development[8-10]. A sensitivity test is performed here to assess the impact on benefits trend and phaseout decision under a more ambitious mitigation trajectory of coal power generation, by incorporating and harmonizing another generation projection trend of Zhang et al., 2023[11] from 2021-2060 (Supplementary Fig. 21a).

The disparity between strategies remains unchanged, despite a significant decrease in CO_2 emissions and health risk related to coal plants (Supplementary Fig. 21b-c). Taking Health strategy for example, the health risks could decrease from 80,200 (CI, 78,600-81,800) to 62,600 (CI, 61,100-64,300) in 2030 in a lower power demand. Meanwhile, the CO_2 emission ratio between Carbon and Health strategy keeps stable in 0.98 within the original scenario and lower power demand. Similarly, the net benefit trends benchmarked against Historical strategy exhibit minor changes (Supplementary Fig. 21d). For example, the changing ratio of median net benefit of Health strategy is only 6% in both scenarios. This is because the overall characteristics of coal power fleet (including existing and new-built units) remain unchanged, even with a slight variation in generation scenarios. This preserves the differences in benefits within each strategy and leads to minimally influence on the result of preference analysis (Supplementary Fig. 22).

Supplementary Text 8. China coal-fired Power plant Emissions Database

China coal-fired Power plant Emissions Database (CPED) is developed and maintained by Tsinghua University and keeps tracking emissions of coal-fired power plants for a 30-year period. Built upon data from Ministry of Ecology and Environment (MEE; unpublished data), CPED offered year-by-year detailed information about activity data, operating status, emission factors, geographical location, end-of-pipe control technology. More details about CPED can be found in Liu et al., 2015[12] and Tong et al., 2018[13]. Previous studies have verified that the magnitude and trends of power emissions in CPED are in good agreement with top-down estimates from satellite measurements.

CPED used here covers 5,553 coal-fired generating units with 947.8 GW (94% of the total installed capacity) in 2018. According to CPED, coal power consumed more than 50% of the total production, accounts for more than 60% of total national power generation, and contributes significantly to the total emission of CO_2 and air pollutants (17.5% of SO_2, 15.6 of NO_x, 3.9% of $PM_{2.5}$, 34.7% of CO_2 in 2018, see Supplementary Table 7). Coal-fired power plants are primarily located in provinces with abundant resources, high demand, developed industries, and convenient transportation (e.g., Jiangsu, Inner Mongolia, Shandong, Shanxi). This distribution overlaps with densely populated areas in China, intensifying the health risks associated with pollutant emissions.

Additionally, CPED also provides information on 127 GW retired coal power capacity, which helps to characterize the prior phaseout practice since the 11th Five-Year-Plan (Supplementary Fig. 4).

Supplementary Text 9. Preprocessing of baseline emission inventory of coal power

Our unit-level power plant database (i.e., CPED) does have some blank information, which typically occur in unit-level operating conditions and emission information. Supplementary Table 5 shows the quantity of blank spots in the original emission database. To ensure data integrity and preserve key patterns, we have established a comprehensive preprocessing scheme for baseline unit-level coal power emission dataset. A step-wised method is designed to impute the missing value of activity-rate parameters, emission factors and pollutant removal efficiency.

For missing basic information and operating conditions, the imputation starts at unit- or plant-level where missing values are filled by the prior values from the same units or units in the same plant. If the first step is not feasible, averaged values form other units with similar installed capacity in the same province are used to fill the blank spots. Validation of imputed values is conducted based on the data type, leveraging sectoral official statistical data. For example, the summation of unit-level coal power generation is cross-validated against provincial-level generation.

The imputation of missing data related to emission factors is similar to the above-mentioned processes, while the dynamic natures of fossil coal (e.g., sulfur content and ash content) are further taken into account by using provincial trends of variation. The imputation of missing pollutants control information is more complicated, which involves a dynamic model considering emission standards and retrofitting progress. For units with available end-of-pipe control information meeting current emission standards, we fill the blank spots by using prior information. In cases of other missing values, the emission standard is used to calculate the emission control efficiency, with priority given to installing end-of-pipe control devices for young units with large installed capacity. The model undergoes iterative processes of self-correction until it aligns with the principle that the imputed end-of-pipe control information (i.e., retrofitting progress and average control level) corresponds with the statistical data.

Supplementary Text 10. Sensitivity test of health risk estimation

Traditional concentration-response functions, which the lack of availability of source-specific exposure estimates, are likely to underestimate mortality burden attributed to coal power[14]. Advanced research based on U.S.A. Medicare beneficiaries reported that exposure to coal $PM_{2.5}$ may lead to a mortality risk approximately 2.1 times greater than exposure to $PM_{2.5}$ from all sources[15]. Limit to the absence of relevant epidemiological data and tools, our study is temporarily unable to employ a source specified health risk assessment tailored to coal power in China. The potential impact of underestimating health risks on the net benefits of each strategy is assessed by conducting additional sensitivity tests on the health risk attributed to Coal $PM_{2.5}$.

The underestimation of health effects does indeed have a noticeable impact (Supplementary Fig. 23). Using the Carbon strategy as an example, the overall net benefits increase from -378 (CI, -528~-231) to 448 (CI, 134~758) billion RMB with a health risk correction factor of 2.1 times, and there is almost no risk of negative outcomes. The implementation of those targeted strategies may yield unexpected magnitude of overall benefits, compared with the historical one.

Limited changed is observed in the preference-based phaseout decision compared to the original scenario (Supplementary Fig. 27). This is because the correction factor on the health risk of coal $PM_{2.5}$ had no impact on the relative rankings and differences of normalized health co-benefit among different phaseout pathways, which is adheres to the principle that health co-benefit might only play a secondary role as indirect outcomes under policy preferences devaluing health considerations.

In response to the call for Healthy China Initiative, we believe that health protection will become a pivotal consideration in future decision-making. There is an urgent need to update the coal-specified health assessment methodology and reduce associated uncertainties.

Supplementary Text 11. Sensitivity test of carbon price and disturbance groups

Since the launch of emission trading system in med-2021, China's carbon price is steady at 40~60 RMB/t CO_2. According to some literatures[16,17], China's carbon price is expected to rise in the future and drive structural changes in energy sectors, which might lead to underestimating the economic benefits of decarbonization from coal power phaseout policy in our study. To evaluate how changes in carbon prices would affect the cost-effectiveness of the phaseout decision, sensitivity tests are conducted based on European carbon price (75.5 US dollars/ t CO_2). All phaseout strategies could obtain a promising positive cumulative net benefit if European carbon price is implemented (Supplementary Fig. 11). This highlights the increasing cost-effectiveness in phaseout policy under ambitious climate goals.

To verify the robustness and practicableness of the model, some configuration in our model needs to be further investigated. As Method mentioned, we disturb the phaseout priorities of each phaseout strategy, after evenly dividing them into 10 groups according to their rank, to simulate the disruption in sorting the phaseout priorities of units with similar characteristic. To evaluate the impact of the number of disturbance groups, we design a verification experiment to compare the overall net benefits of each strategy under different number of disturbance groups (i.e., 5, 10, 20). The impact of grouping numbers on the main analyses and findings in this study is limited. (See Supplementary Fig. 28). Therefore, the disturbed phaseout priorities could be used as a reliable measure to simulate the disturbance in policy implementation.

Supplementary Text 12. Uncertainty decomposition

We quantify the contribution from two drivers to the uncertainty of annual net benefits of each strategy, including phaseout of existing units and construction of new-built units here. In our study, single-factor experiments are applied to uncertainty decomposition. Under specific strategy, we first establish a fixed phaseout order which comes from the pathway with median cumulative benefits, and maintain a random disruption in the priority of new unit construction for all pathways. This enables us to determine the uncertainty range (CI, defined as 95% confidence interval) attributed to construction of new-built units. Next, we establish a fixed order of new-built units which comes from the pathway with median cumulative benefits, and maintain a random disruption in the phaseout priority for all pathways. This enables us to determine the uncertainty range attributed to the disturbance in phaseout decision.

Based on the above two uncertainty range, the annual uncertainty of net benefits is decomposed as equation (1)-(4) below:

$$sf_{old,y,i} = \frac{CI_{old,y,i}}{CI_{new,y,i} + CI_{old,y,i}} \quad (1)$$

$$sf_{new,y,i} = \frac{CI_{new,y,i}}{CI_{new,y,i} + CI_{old,y,i}} \quad (2)$$

$$CI^{*}_{old,y,i} = CI_{origin,y,i} * sf_{old,y,i} \quad (3)$$

$$CI^{*}_{new,y,i} = CI_{origin,y,i} * sf_{new,y,i} \quad (4)$$

Where *y, i* represents specific year and specific phaseout strategy; $CI_{origin,y}$ represents the uncertainty range of phaseout strategy *i*; $CI_{old,y,i}$ and $CI_{new,y,i}$ represent the uncertainty range of single-factor experiments for two drivers (i.e., phaseout of existing units and construction of new-built units), respectively; $sf_{old,y,i}$ and $sf_{new,y,i}$ represent the scale factors to decompose the overall annual uncertainty; $CI^{*}_{old,y,i}$ and $CI^{*}_{new,y,i}$ represent the uncertainty ranges of net benefits from strategy *i* in year *y* attributed to phaseout of existing units and construction of new-built units, respectively.

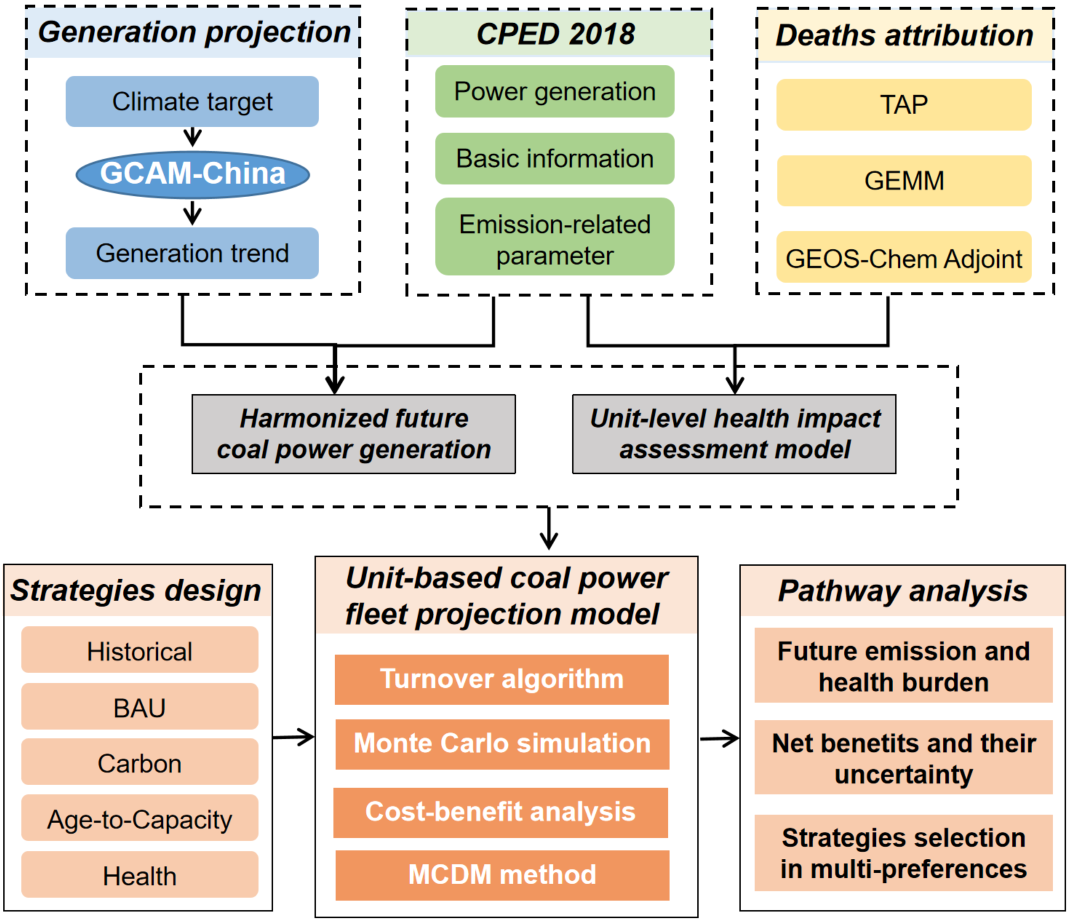

Supplementary Figure 1 | Roadmap of detailed modeling framework. This figure shows the modeling framework of cost-effectiveness uncertainty assessment and preference-based decision making for coal power transition in China.

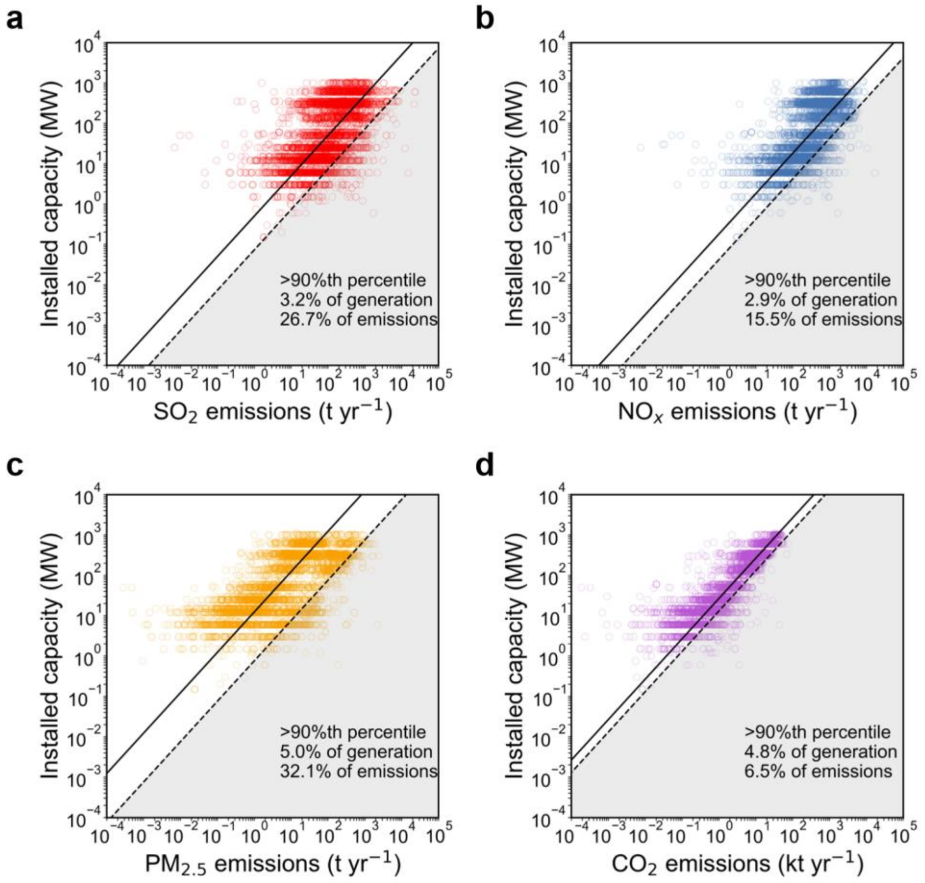

Supplementary Figure 2 | The distribution of unit-level emission intensity. Points represent individual coal-fired units, in each case plotted according to nameplate capacity (y-axis) and annual emissions of SO_2 **(a)**, NO_x **(b)**, $PM_{2.5}$ **(c)** and CO_2 **(d)** (x-axis). Emission intensity is defined as CO_2 or pollutants emissions per capacity. Solid diagonal lines indicate the median emission intensity of each species and shaded triangles indicate units whose emission intensity is over 90th percentile.

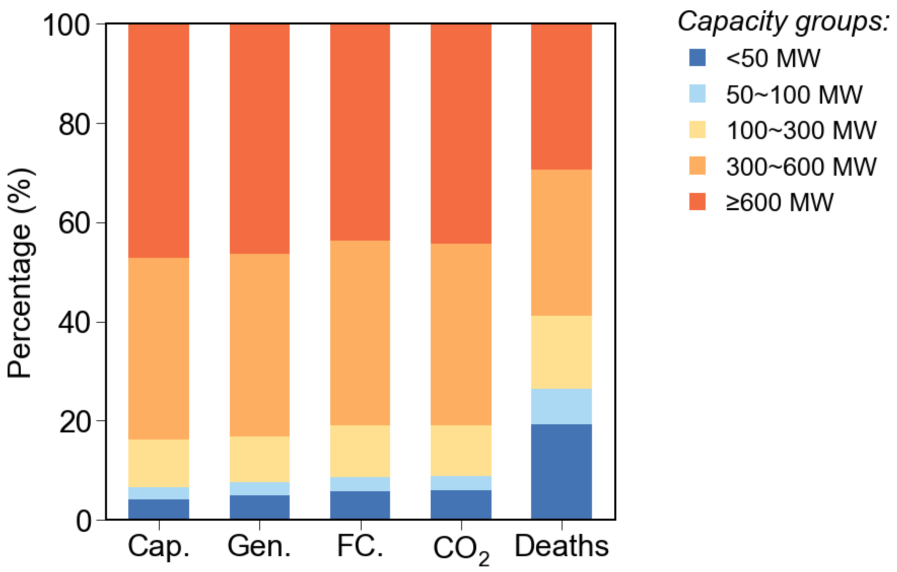

Supplementary Figure 3 | Shares of total capacity and related impacts by capacity groups. Bars from left to right show the fraction of capacity (Cap.), generation (Gen.), fuel consumptions (FC.), CO_2 emissions (CO_2), and premature deaths (Deaths) caused by units in five capacity groups.

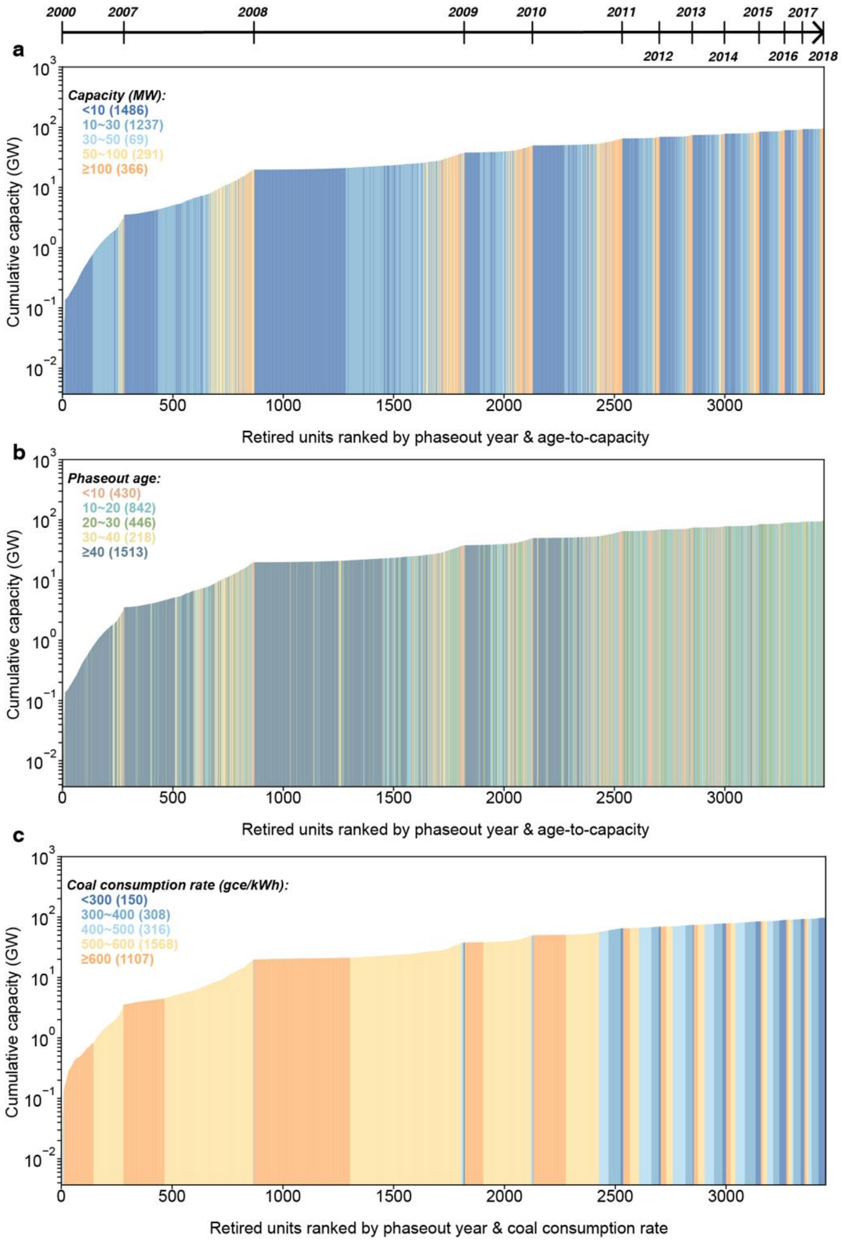

Supplementary Figure 4 | Cumulative capacity by individual retired units for coal power, ranked by the phaseout year and ratio of Age-to-Capacity or coal consumption rate. (a-b) The bar represents the cumulative capacity of individual generating units in order, ranking by both phaseout year and ratio of age to capacity. **(a)** bars are distinguished by capacity groups, **(b)** bars are distinguished by age groups. **(c)** is similar to **(a)** and **(b)**, but units are ranked by both phaseout year and coal consumption rate, and distinguished by coal consumption rate groups.

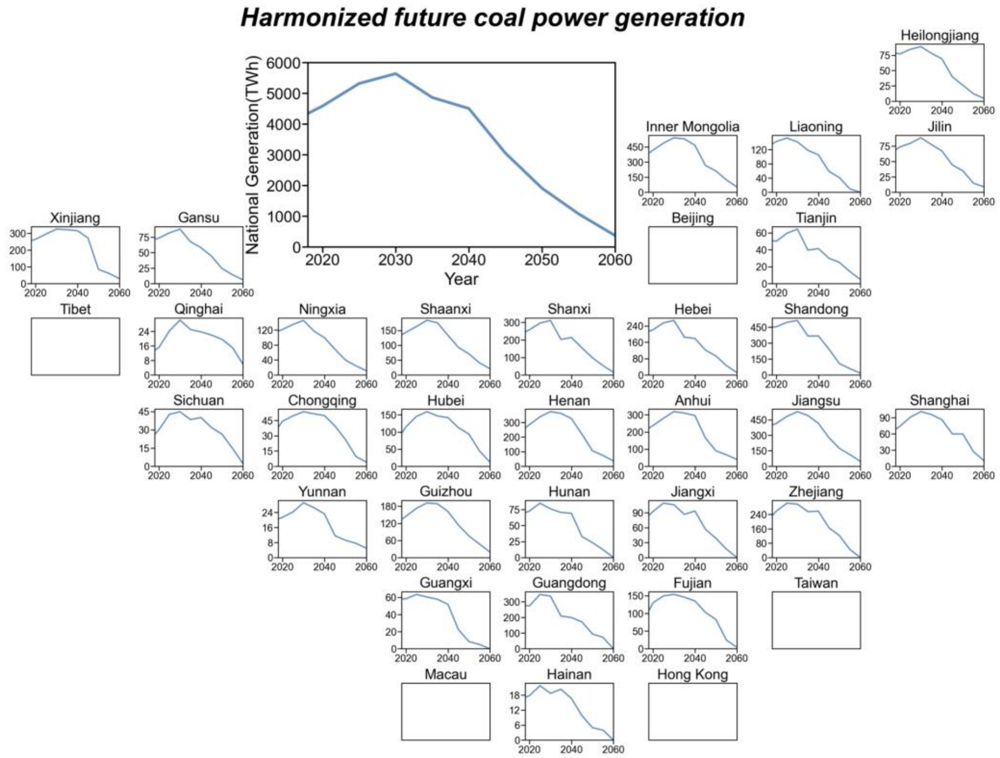

Supplementary Figure 5 | Harmonized national and provincial coal power demand projection. Solid lines represent the projection of coal power demand in line with carbon peak and carbon neutrality goals. The subfigure with bigger size represents the national coal power demand projection and other subfigures represent the provincial coal power demand projection.

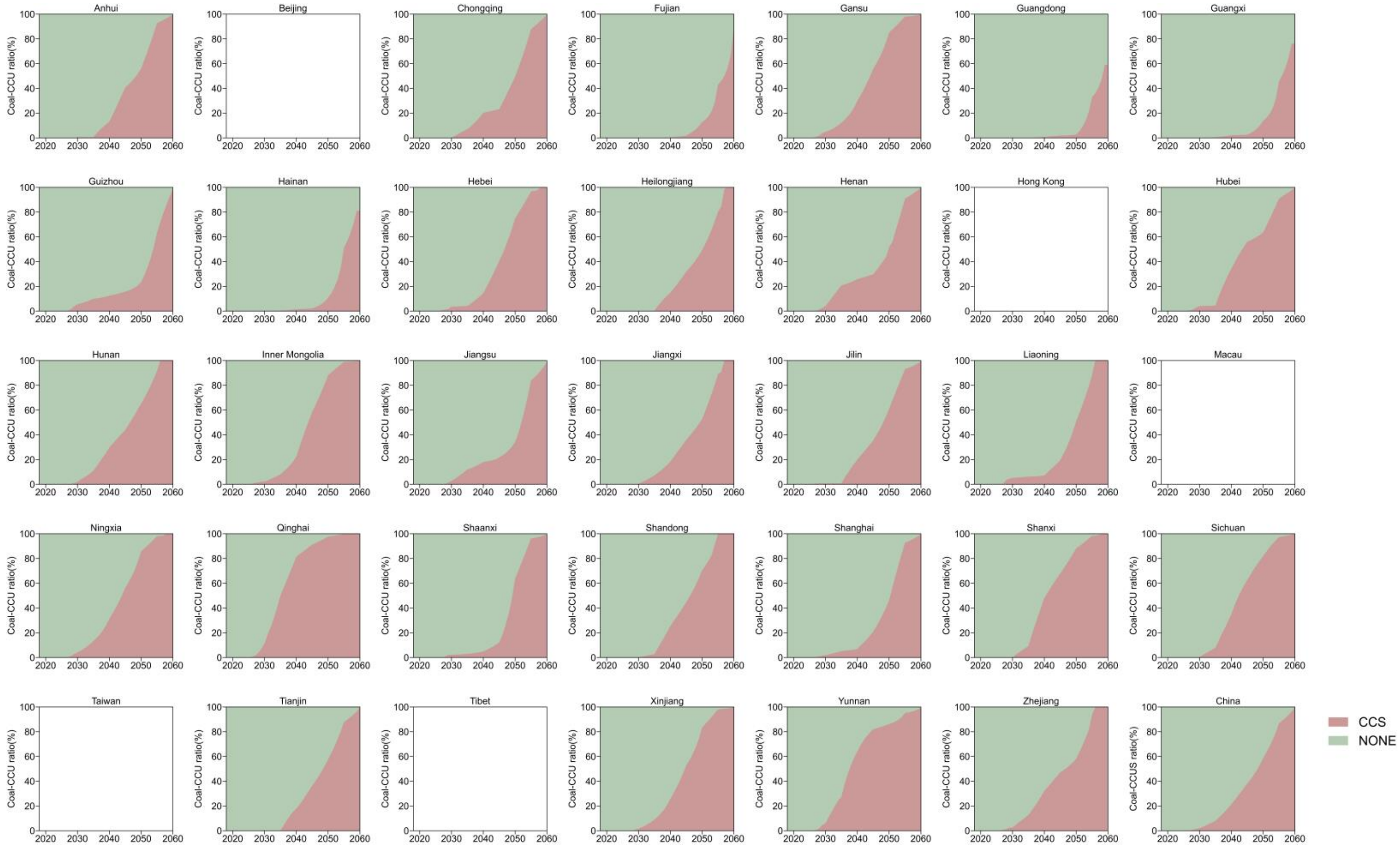

Supplementary Figure 6 | Penetration rates of Carbon Capture, Utilization, and Storage (CCUS) in coal-fired power units. The pink stacked areas represent the national and provincial CCUS penetration rates in line with carbon peak and carbon neutrality goals, while the green stacked areas represent the national and provincial penetration rates of coal power units without CCUS retrofitting.

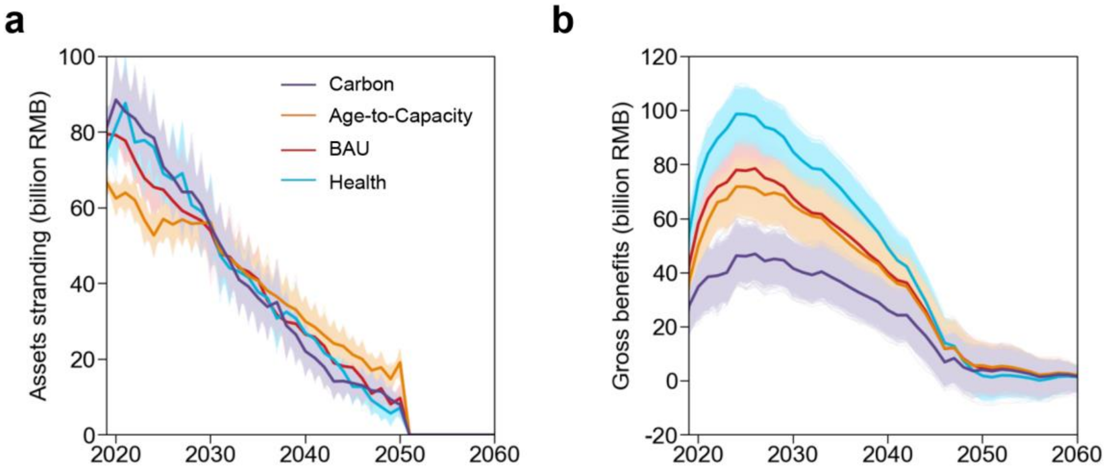

Supplementary Figure 7 | Trends in and possible ranges of assets stranding and gross benefits for each strategy. (a) Assets stranding. **(b)** Gross benefits compared to the Historical strategy. The solid lines and the shaded areas represent the median value and the uncertainty of each indicator under each strategy, respectively.

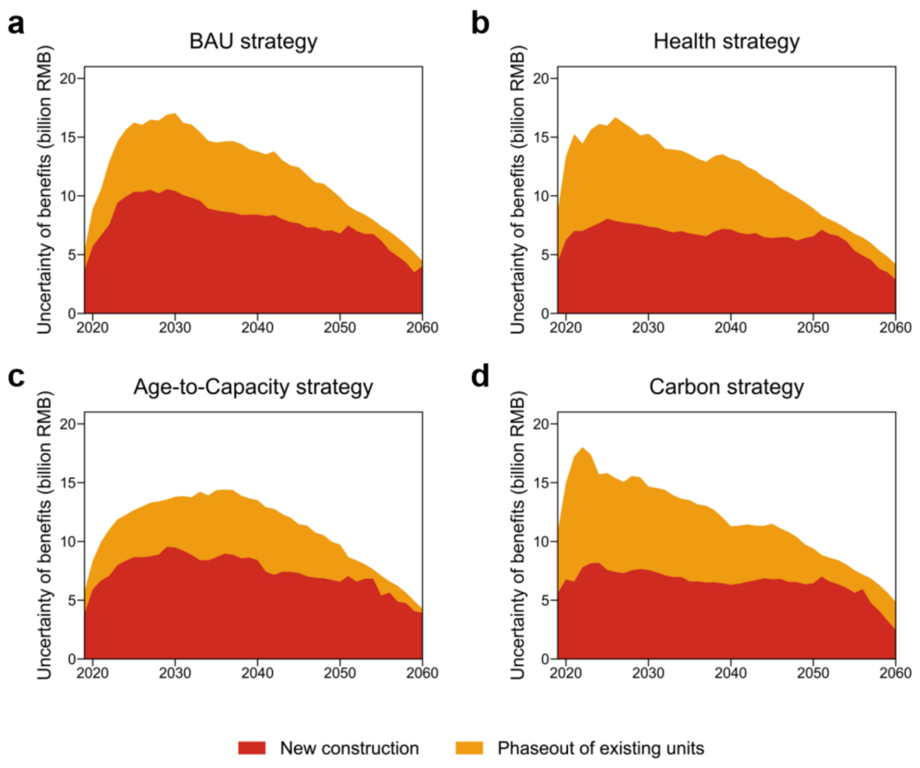

Supplementary Figure 8 | Decomposition of uncertainty in net benefits trend of each coal power transition strategy. (a) The stacked areas show the decomposition of cost-effectiveness uncertainty of BAU (business as usual) strategy. **(b)** The stacked areas show the decomposition of cost-effectiveness uncertainty of Health strategy. **(c)** The stacked areas show the decomposition of cost-effectiveness uncertainty of Age-to-Capacity strategy. **(d)** The stacked areas show the decomposition of cost-effectiveness uncertainty of Carbon strategy. The cost-effectiveness uncertainty is attributed to both the introduction of new capacity (red) and the phaseout of existing units (orange).

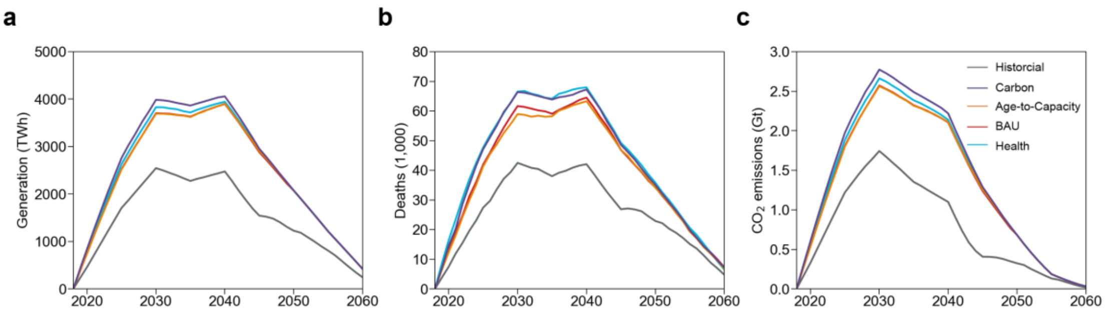

Supplementary Figure 9 | Trends of coal generation, deaths, CO_2 emissions of new-built capacity across each strategy. (a) Coal power generation. **(b)** Coal power-related premature deaths **(c)** CO_2 emissions. The solid lines represent the median value of each indicator under each strategy.

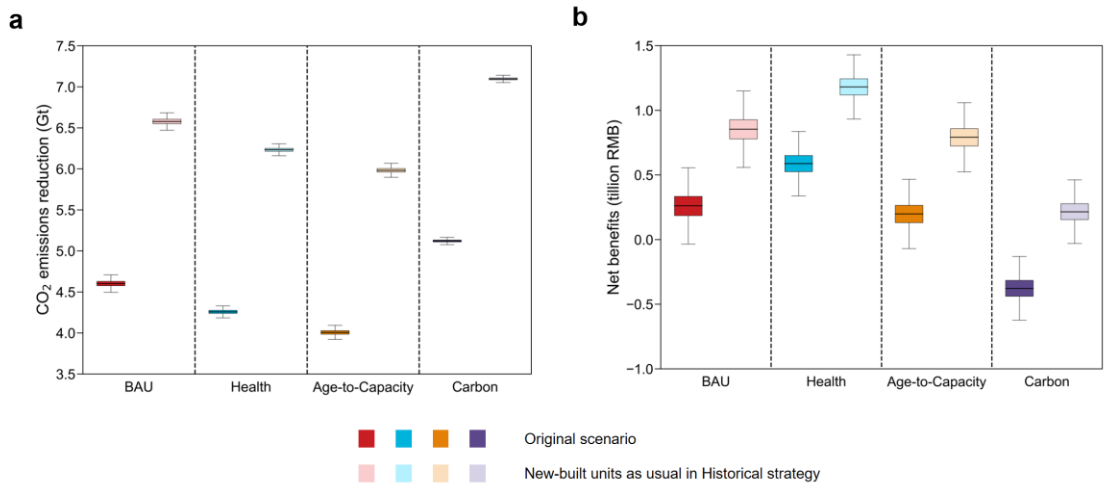

Supplementary Figure 10 | Comparison of the cumulative CO_2 emission reduction and net benefits under original scenario and baseline test considering technological advancement. **(a)** CO_2 emissions reduction. **(b)** Net benefits compared to the Historical strategy. The boxes show the distribution (i.e., the range from the 25th to 75th percentiles) of each indicator under each strategy in original scenario (dark colors) and baseline test considering technological advancement (light colors); the black lines represent the median values.

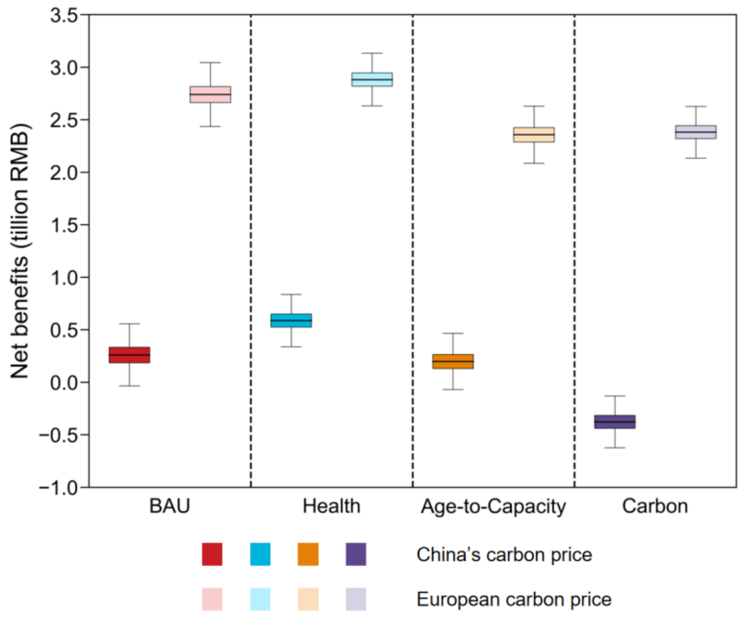

Supplementary Figure 11 | Comparison of the cumulative net benefits under carbon prices in the China's emission trading system and the European emission trading system. The boxes show the distribution (i.e., the range from the 25th to 75th percentiles) of cumulative net benefits under carbon prices in the China's emission trading system (dark colors) and the European emission trading system (light colors); the black lines represent the median values.

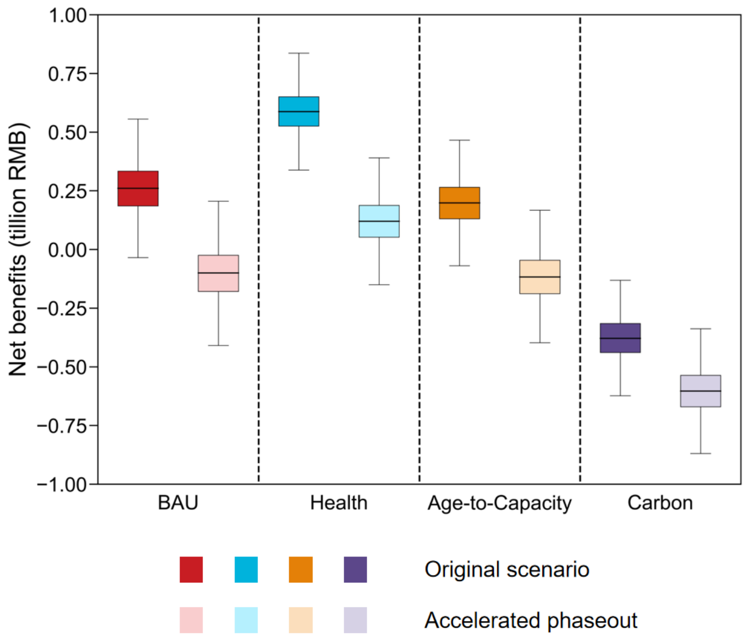

Supplementary Figure 12 | Comparison of the cumulative net benefits under original and accelerated phaseout rate. The boxes show the distribution (i.e., the range from the 25th to 75th percentiles) of cumulative net benefits under original (dark colors) and accelerated phaseout rate (light colors); the black lines represent the median values.

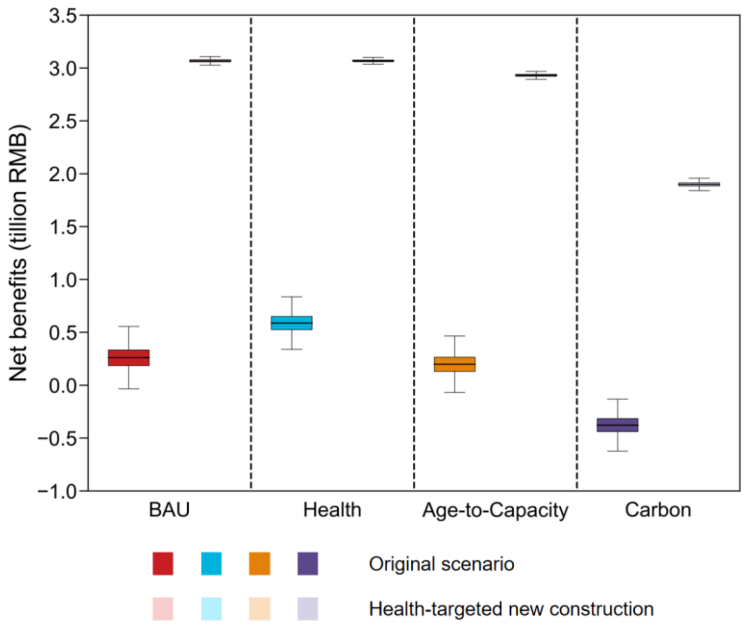

Supplementary Figure 13 | Comparison of the cumulative net benefits under random site selection and strategic site selection of new-build units. The boxes show the distribution (i.e., the range from the 25th to 75th percentiles) of cumulative net benefits under random site selection (dark colors) and strategic site selection (light colors) of new-build units; the black lines represent the median values.

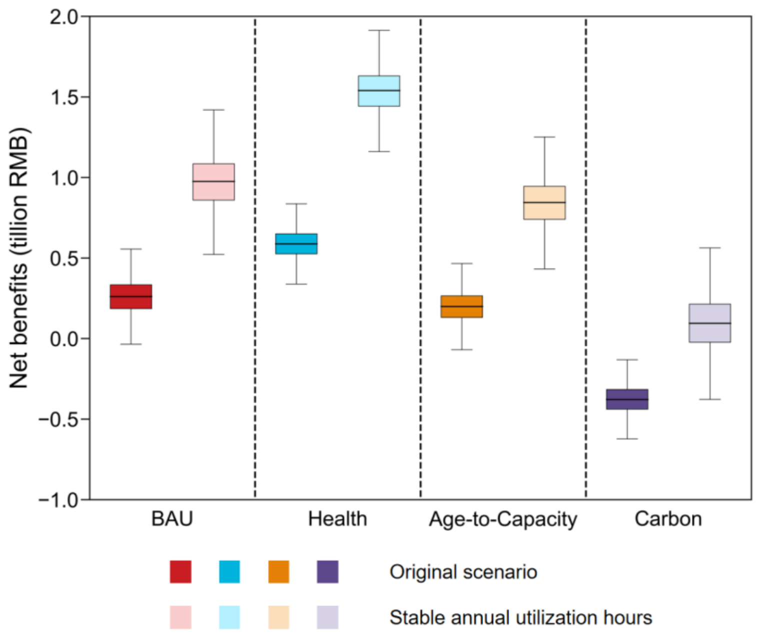

Supplementary Figure 14 | Comparison of the cumulative net benefits under original scenario and sensitivity test assuming stable annual utilization hours for all units. The boxes show the distribution (i.e., the range from the 25th to 75th percentiles) of cumulative net benefits under original scenario (dark colors) and sensitivity test assuming stable annual utilization hours for all units (light colors); the black lines represent the median values.

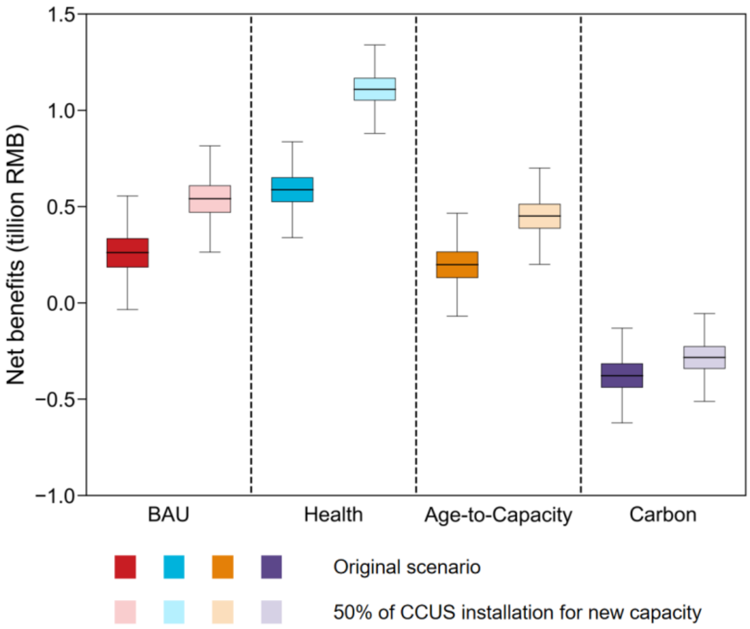

Supplementary Figure 15 | Comparison of the cumulative net benefits under original scenario (i.e., CCUS would be prioritized for new-built capacity) and sensitivity test (allocating 50% of the CCUS demand to new-built units and assign the remaining demand to existing units). The boxes show the distribution (i.e., the range from the 25th to 75th percentiles) of cumulative net benefits under original scenario (i.e., CCUS would be prioritized for new-built capacity, dark colors) and sensitivity test (allocating 50% of the CCUS demand to new-built units and assign the remaining demand to existing units, light colors); the black lines represent the median values.

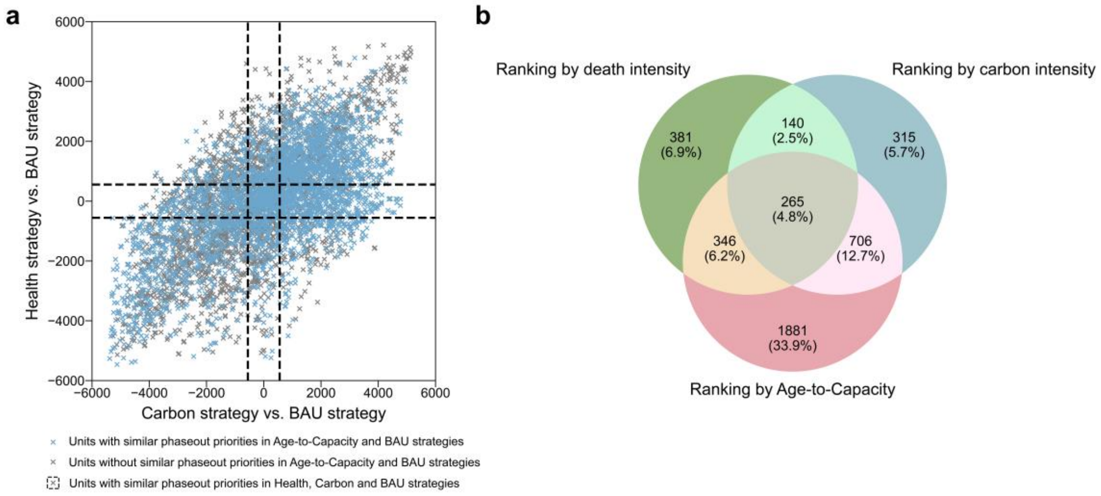

Supplementary Figure 16 | Differences in phaseout priorities between targeted strategies and BAU (business as usual) strategy. (a) The unit-level phaseout priorities difference between targeted strategies and BAU strategy. The dots in central square area represent units with similar phaseout priorities (ranking difference<500) in Health, Carbon and BAU strategies. The blue dots represent units with similar phaseout priorities in both Age-to-Capacity and BAU strategies and the grey dots represent units without similar phaseout priorities in both Age-to-Capacity and BAU strategies. **(b)** Venn diagrams showing the numbers of shared and unique units with similar phaseout priorities between targeted strategies and BAU strategy.

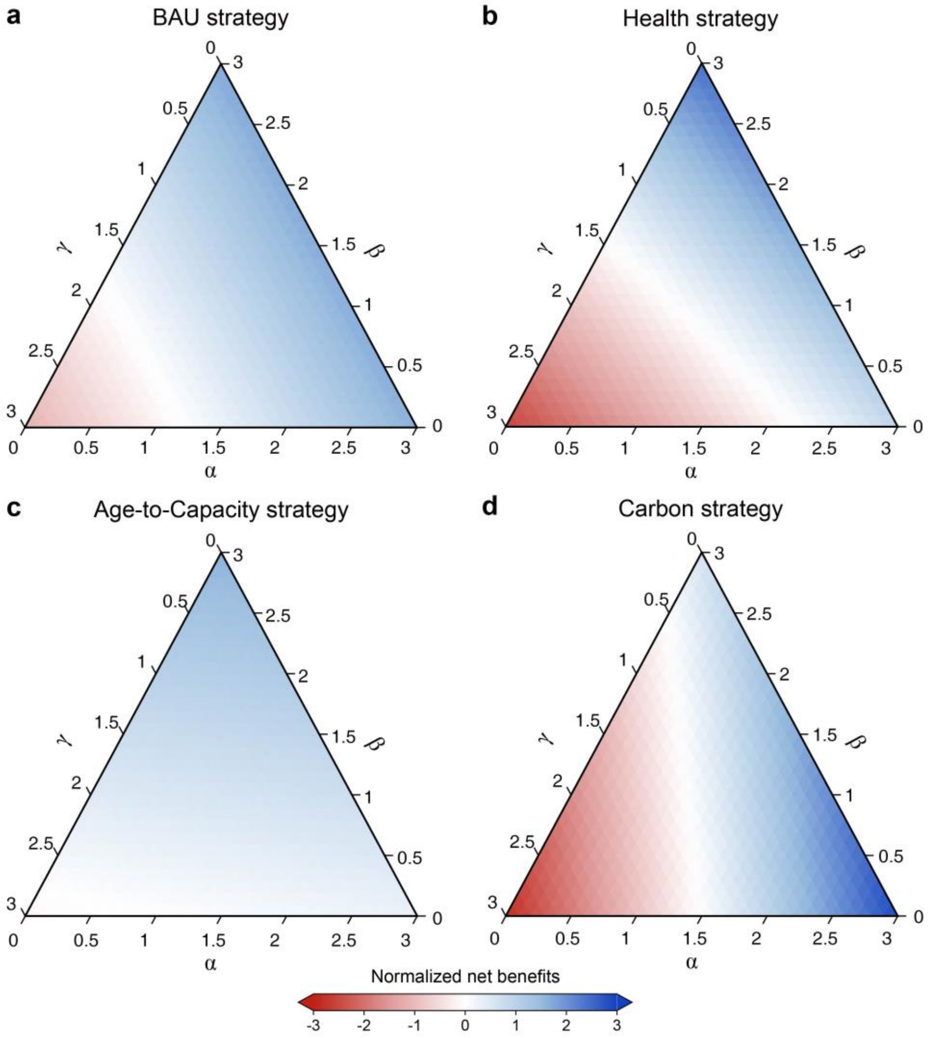

Supplementary Figure 17 | Normalized total benefits of each strategy under different decision preferences. (a) Contours show the preference-based normalized net benefits of BAU (business as usual) strategy. **(b)** Contours show the preference-based normalized net benefits of Health strategy. **(c)** Contours show the preference-based normalized net benefits of Age-to-Capacity strategy. **(d)** Contours show the preference-based normalized net benefits of Carbon strategy. α, β and γ represent the preference weighting factor of climate change mitigation, public health protection and assets stranding avoiding, respectively. The higher value of the specific preference weighting factor, the more emphasis is placed on the that preference.

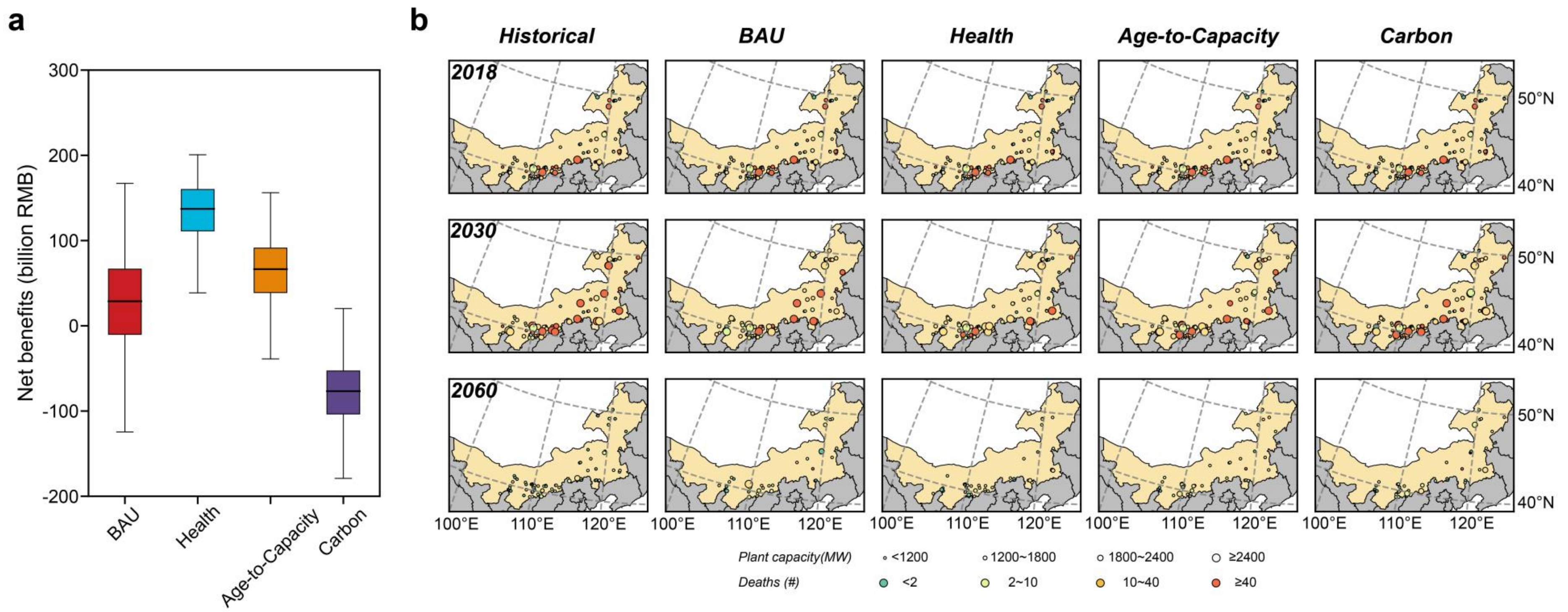

Supplementary Figure 18 | Cumulative net benefits and the potential turnover of power fleet for each strategy in Inner Mongolia. (a) The boxes show the distribution (i.e., the range from the 25th to 75th percentiles) of cumulative net benefits under each strategy and the black lines represent the median values. Cumulative net benefits of targeted strategies represent the sum of monetized CO_2 emission reduction benefits and health co-benefits minus the costs of asset stranding. **(b)** The maps show the location and evolution of coal-fired power plants in 2018, 2030, and 2060 for each strategy, respectively. Plants are classified by installed capacity and health risks.

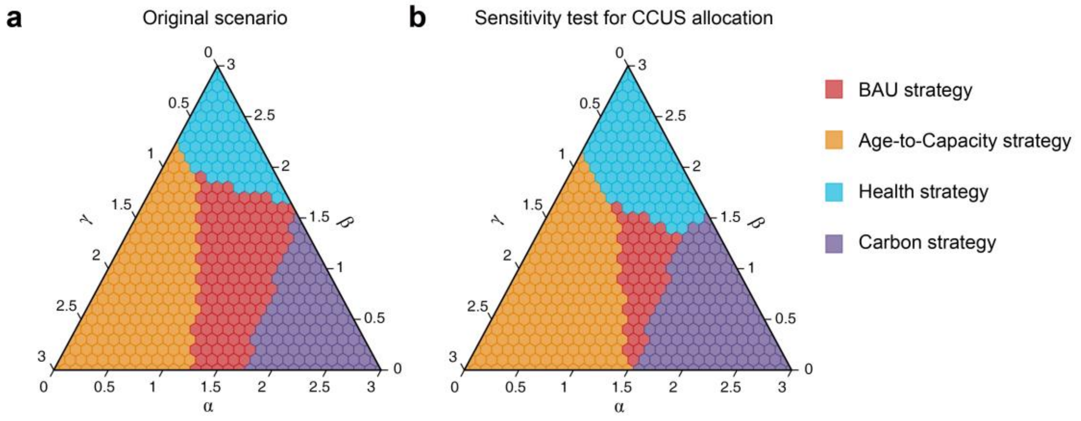

Supplementary Figure 19 | Preference analysis for coal power phaseout strategy design. **(a)** Coal power phaseout strategy selection according to the maximum normalized total benefits under the original scenario (i.e., CCUS would be prioritized for new-built capacity). **(b)** Coal power phaseout strategy selection according to the maximum normalized total benefits under the sensitivity test (allocating 50% of the CCUS demand to new-built units and assign the remaining demand to existing units). α, β and γ represent the preference weighting factor of climate change mitigation, public health protection and assets stranding avoiding, respectively. The higher value of the specific preference weighting factor, the more emphasis is placed on the that preference.

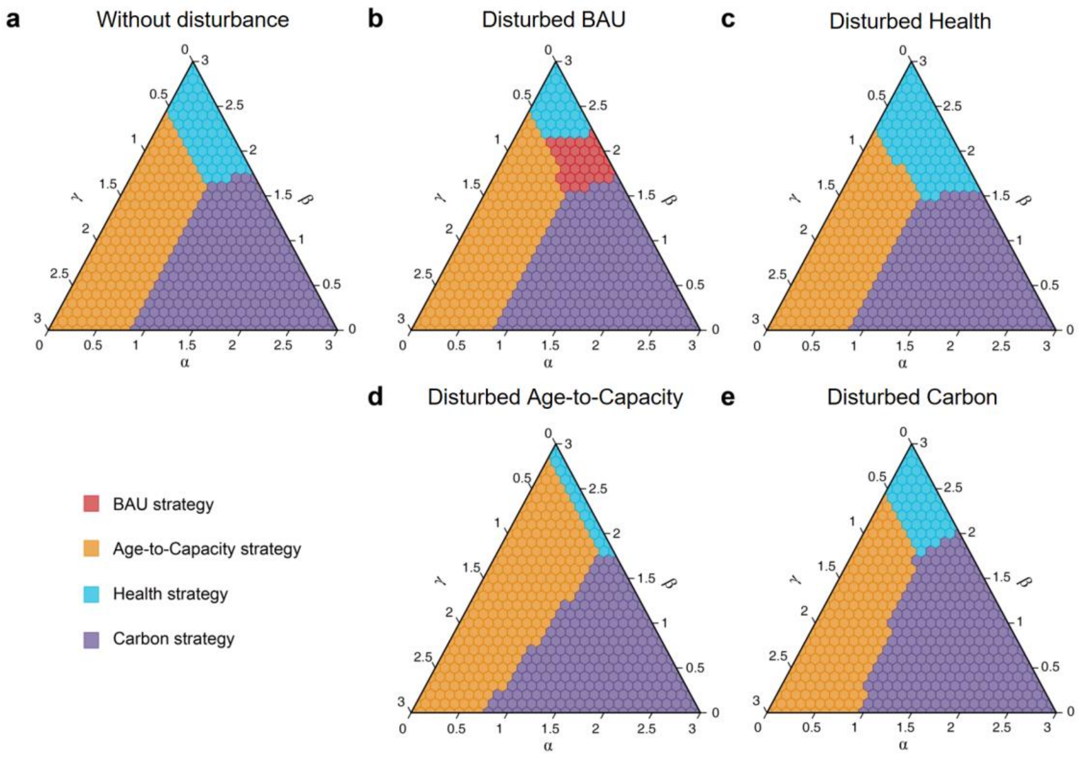

Supplementary Figure 20 | Preference analysis for coal power phaseout strategy design tailored to Inner Mongolia. (a) Coal power phaseout strategy selection according to the maximum normalized total benefits. α, β and γ represent the preference weighting factor of climate change mitigation, public health protection and assets stranding avoiding, respectively. The higher value of the specific preference weighting factor, the more emphasis is placed on the that preference. **(b-e)** Disturbed phaseout strategy decisions, which are disturbed by the pathway with 95th percentile of monetized net benefits under BAU **(b)**, Health **(c)**, Age-to-Capacity **(d)**, and Carbon **(e)** strategies, respectively.

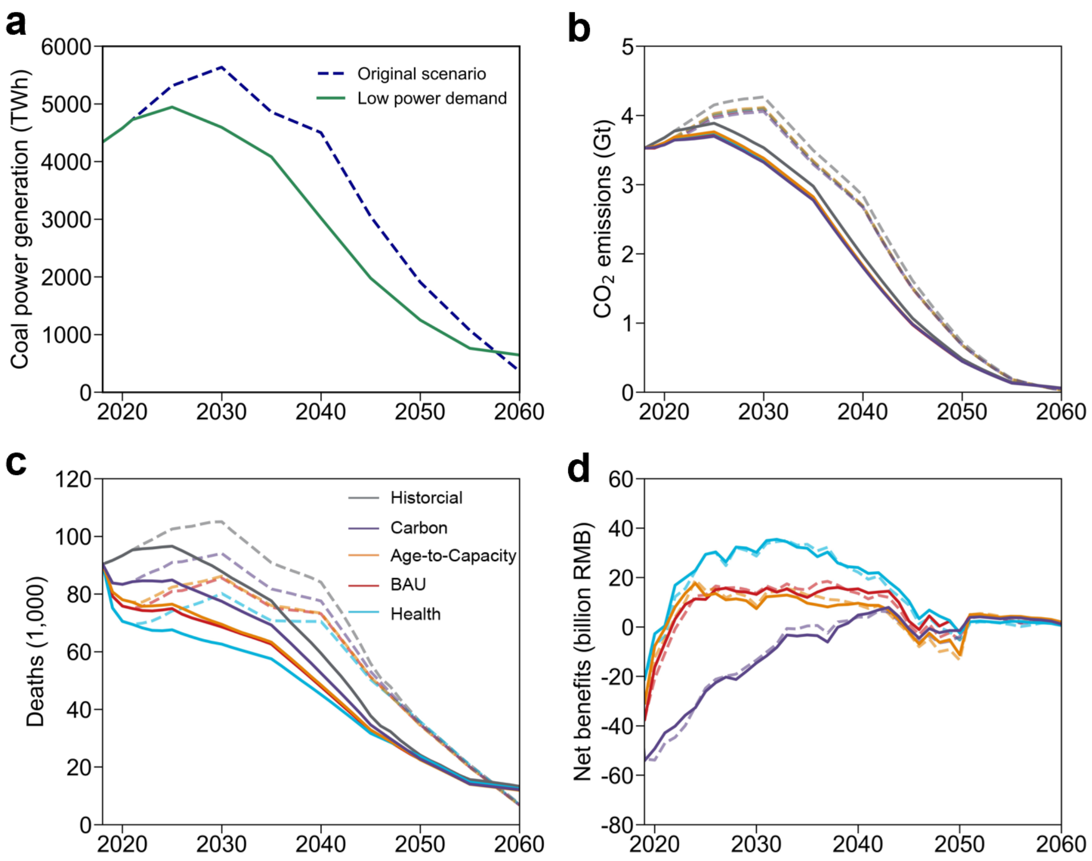

Supplementary Figure 21 | Trends of coal power generation, CO_2 emissions, deaths, and benefits for each strategy under different mitigation trajectory. (a) Projection of coal power generation in China **(b)** CO_2 emissions**. (c)** Coal-power-related premature deaths. **(d)** Monetized benefits compared to the historical phaseout pathway. The lines represent the median value of each indicator within each strategy under the original scenario (solid lines) and sensitivity test of lower power demand (dashed lines).

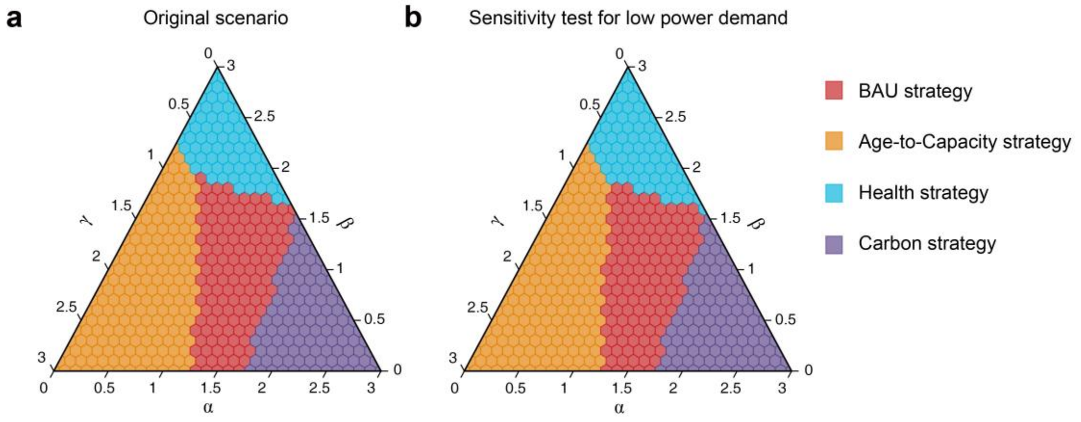

Supplementary Figure 22 | Preference analysis for coal power phaseout strategy design. Coal power phaseout strategy selection according to the maximum normalized total benefits under original scenario (**a**) and sensitivity test of lower power demand (**b**). α, β and γ represent the preference weighting factor of climate change mitigation, public health protection and assets stranding avoiding, respectively. The higher value of the specific preference weighting factor, the more emphasis is placed on the that preference.

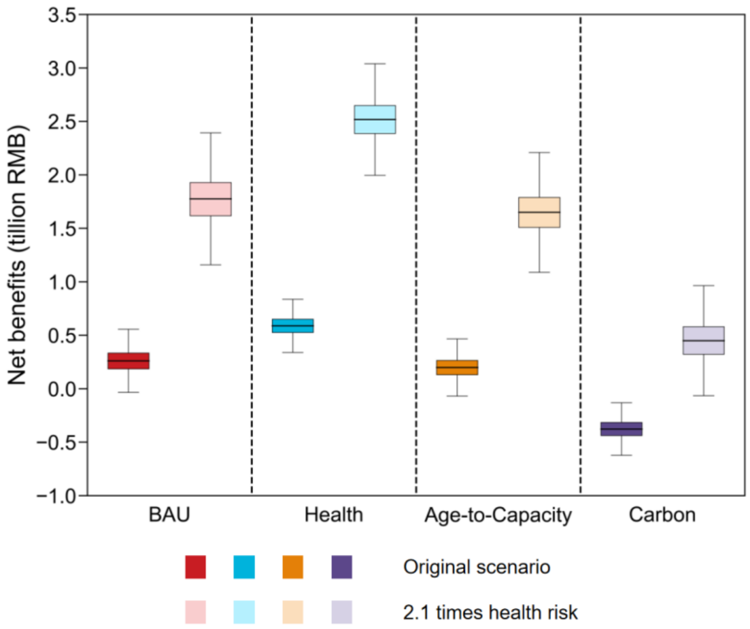

Supplementary Figure 23 | Comparison of the cumulative net benefits under original and 2.1 times health risk. The boxes show the distribution (i.e., the range from the 25th to 75th percentiles) of cumulative net benefits under original scenario (dark colors) and sensitivity test with 2.1 times health risk (light colors); the black lines represent the median values.

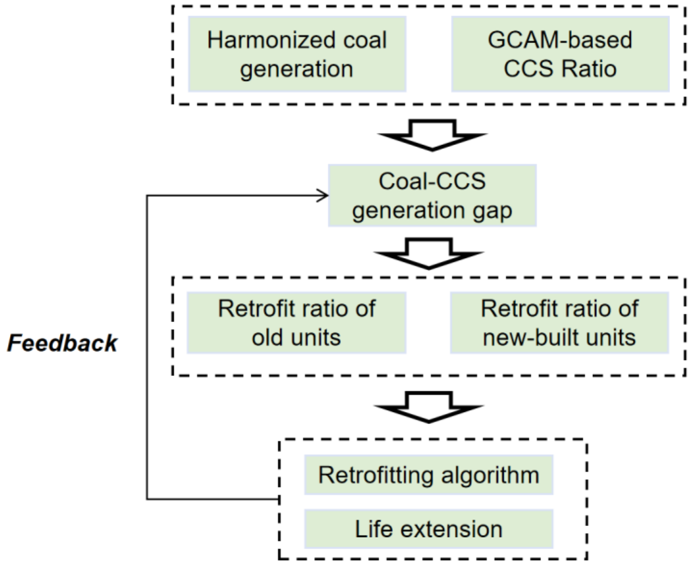

Supplementary Figure 24 | Method framework of CCUS retrofitting in coal-fired power units. This figure shows the method framework of CCUS retrofitting and life extension constrained by harmonized provincial coal power demand projection and the CCUS retrofitting ratio.

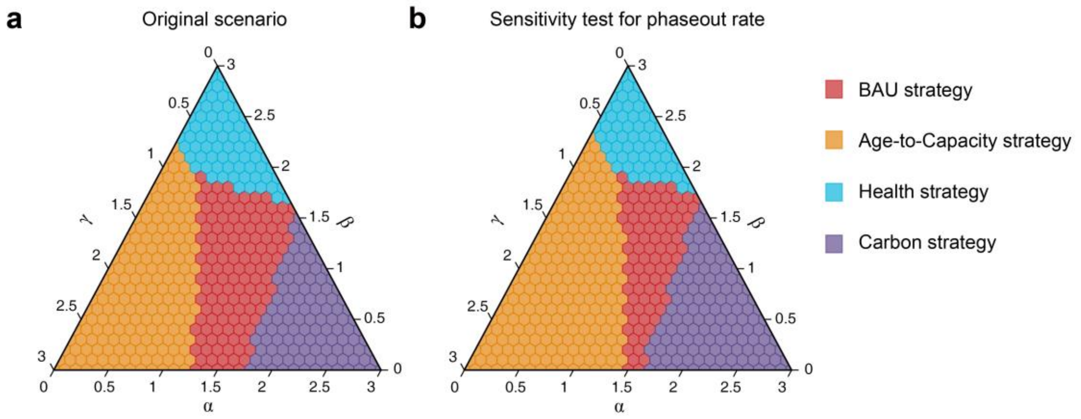

Supplementary Figure 25 | Preference analysis for coal power phaseout strategy design. Coal power phaseout strategy selection according to the maximum normalized total benefits under original phaseout rate **(a)** and accelerated phaseout rate **(b)**. *α*, *β* and *γ* represent the preference weighting factor of climate change mitigation, public health protection and assets stranding avoiding, respectively. The higher value of the specific preference weighting factor, the more emphasis is placed on the that preference.

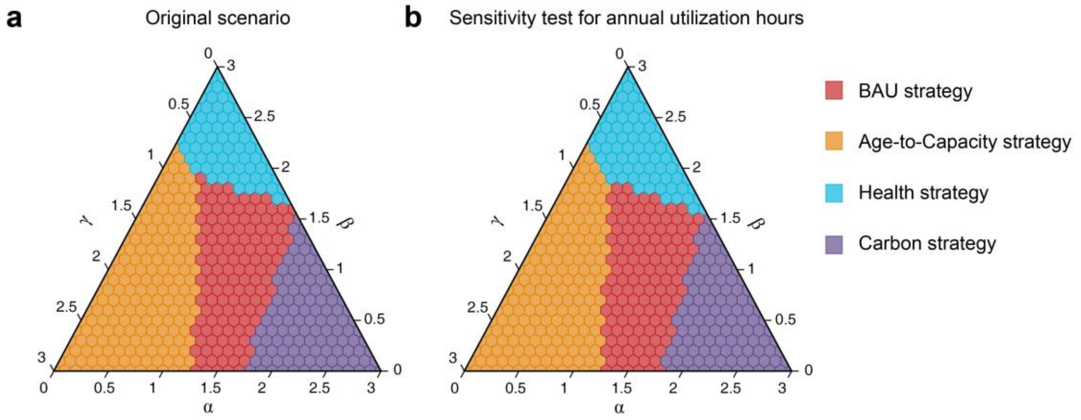

Supplementary Figure 26 | Preference analysis for coal power phaseout strategy design. Coal power phaseout strategy selection according to the maximum normalized total benefits under original scenario **(a)** and sensitivity test assuming stable annual utilization hours for all units **(b)**. α, β and γ represent the preference weighting factor of climate change mitigation, public health protection and assets stranding avoiding, respectively. The higher value of the specific preference weighting factor, the more emphasis is placed on the that preference.

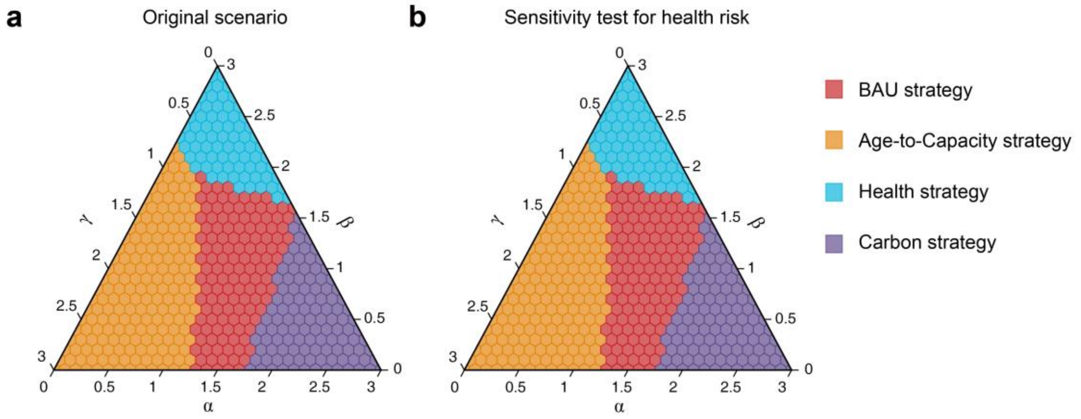

Supplementary Figure 27 | Preference analysis for coal power phaseout strategy design. Coal power phaseout strategy selection according to the maximum normalized total benefits under original (**a**) and 2.1 times health risk (**b**). *α*, *β* and *γ* represent the preference weighting factor of climate change mitigation, public health protection and assets stranding avoiding, respectively. The higher value of the specific preference weighting factor, the more emphasis is placed on the that preference.

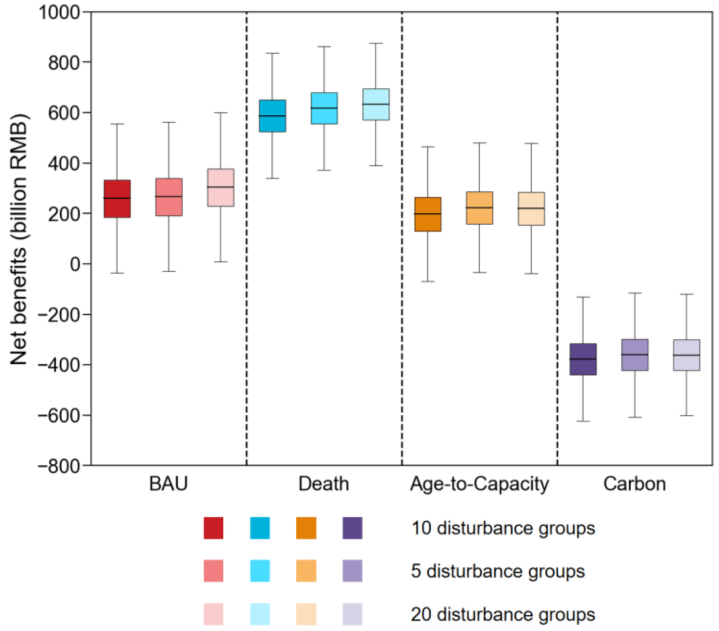

Supplementary Figure 28 | Sensitivity testing for number of disturbance groups. The boxes from left to right show the distribution (i.e., the range from the 25th to 75th percentiles) of cumulative net benefits under original scenario (separating all units into 10 disturbance groups according to phaseout priorities) and two sensitivity tests which separate all units into 5 and 20 disturbance groups according to phaseout priorities, respectively; the black lines represent the median values.

Supplementary Table 1 | The coal power transition strategies.

Coal power transition strategy	Objective	Phaseout and new-built roles
Historical strategy	Reflecting the economic consideration of return on investment	The 40-year lifetime is set for all units. Units that have been operating for more than 40 years in 2018 would phaseout evenly within 10 years.<br>The new-built units are randomly constructed on the site of retired units.
BAU strategy	Following prior phaseout practice	Units with low value of survival probability are prioritized for phaseout, according to the survival curve of Cox model.<br>A 32% margin of disturbance is set in the phaseout priority and the new-built units are randomly constructed on the site of retired units.
Carbon strategy	Deep decarbonization	Units with large value of carbon intensity are prioritized for phaseout.<br>A 32% margin of disturbance is set in the phaseout priority and the new-built units are randomly constructed on the site of retired units.
Health strategy	Public health protection	Units with large value of death intensity are prioritized for phaseout.<br>A 32% margin of disturbance is set in the phaseout priority and the new-built units are randomly constructed on the site of retired units.
Age-to-Capacity strategy	Economic loss avoidance	Units with large ratio of age to capacity size are prioritized for phaseout.<br>A 32% margin of disturbance is set in the phaseout priority and the new-built units are randomly constructed on the site of retired units.

Supplementary Table 2 | Baseline tests, sensitivity tests, and case study.

Test group	Assumption
Baseline test for efficiency of new-built capacity in Historical strategy	Coal consumption rate of new capacity in Historical strategy: 308 gce/kWh
Sensitivity test for future phaseout rate	Lifespans of the in-fleet units in Historical phaseout strategy: 30 years<br>Phaseout speed of strategies other than Historical: 40% in 2025, 100% in 2040
Sensitivity test for future coal power demand	Incorporating and harmonizing another generation projection trend of Zhang et al., 2023[11] into the modelling framework
Sensitivity test for annual utilization hours	Removing the assumption of a year-by-year decrease of 2.5% in capacity factor
Sensitivity test for CCUS priority	Allocating 50% of the CCUS demand to new-built units and assign the remaining demand to existing units
Sensitivity test for health risk estimation	Applying a correction factor of 2.1 in the estimation of health risk
Sensitivity test for carbon price	Carbon prices in European emission trading system: 75.5 USD/t CO_2
Sensitivity test for health-targeted site selection of new-build units	Prioritizing new construction on the site with lower health risks according to Geos-Chem adjoint sensitivity analysis
Sensitivity test for disturbance groups	Setting different number of disturbance groups (i.e., 5, 10, 20)
Case study of Inner Mongolia	Same assumption as original scenario

Supplementary Table 3 | The parameters and settings related to cost effectiveness assessment.

**Category**	**Parameters and values**
Overnight capital cost	4,166 RMB/kW
Carbon price	50 RMB/t CO_2

Supplementary Table 4 | Value of Statistical Life in 30 provinces.

Region	VSL (million RMB)
Beijing	4.64
Tianjin	4.562
Hebei	2.876
Shanxi	2.612
Inner Mongolia	3.648
Liaoning	3.848
Jilin	3.21
Heilongjiang	3.212
Shanghai	4.924
Jiangsu	3.718
Zhejiang	3.704
Anhui	2.648
Fujian	3.568
Jiangxi	2.678
Shandong	3.514
Henan	2.706
Hubei	2.922
Hunan	2.788
Guangdong	3.724
Guangxi	2.446
Hainan	2.62
Chongqing	2.926
Sichuan	2.662
Guizhou	1.968
Yunnan	2.25
Shaanxi	2.704
Gansu	2.238
Qinghai	2.574
Ningxia	2.426
Xinjiang	2.544

Supplementary Table 5. Ratio of blank spots in CPED

Data type	Parameter	Ratio of blank spots
Basic information	Installed capacity	0.2%
	Operating time	0.5%
Operating conditions	Capacity factor	2.2%
	Generation	2.1%
	Coal consumption	1.7%
Emission information	Emission factor	3.4%
	Pollutants control rate	4.7%

Supplementary Table 6 | The parameters and setting related to future turnover.

Module	Sub category	Parameters and values
Phaseout of in-fleet units	Lifespans of the in-fleet units in Historical phaseout strategy	40 years
	Phaseout speed of strategies other than Historical strategy	2030: 40%, 2050:100%
	Disturbance of phaseout policy implementation	33%
Installation of new-built units	Combustion technology and efficiency of new-built units	Ultra-supercritical: 270 gce/kWh
	Removal efficiency of de-SO_2, de-NO_x, and de-PM device in new-built units	η_{SO_2} : 95%, η_{NO_x} : 85%, $\eta_{PM_{2.5}}$: 99.3%

Supplementary Table 7 | Emission and key parameters of China's coal power plants fleet in 2018.

**Category**	**Subcategory**	**2018**
Activity data	Coal consumption (Mt)	2022.2
	Power generation (TWh)	4312.6
	Coal consumption rate (gce/kWh)	307.6
Capacity sizes	<100 MW	6.5%
	100~300 MW	9.6%
	300~600 MW	36.7%
	⩾600 MW	47.1%
Average removal efficiency	De-SO_2 device	94.0%
	De-NO_x device	73.0%
	De-$PM_{2.5}$ device	98.9%
Emission	SO_2 (Tg/yr)	1.65
	$PM_{2.5}$ (Tg/yr)	0.26
	NO_x (Tg/yr)	3.33
	CO_2 (Tg/yr)	3525.4

References:

1 NDRC & NEA. Notice on carrying out nationwide retrofitting and upgrading of coal-fired power units. (National Development and Reform Commission; National Energy Administration, 2021).

2 Davis, S. J. & Socolow, R. H. Commitment accounting of CO_2 emissions. *Environmental Research Letters* **9** (2014).

3 Tong, D. *et al.* Health co-benefits of climate change mitigation depend on strategic power plant retirements and pollution controls. *Nature Climate Change* **11**, 1077-1083 (2021).

4 Jewell, J., Vinichenko, V., Nacke, L. & Cherp, A. Prospects for powering past coal. *Nature Climate Change* **9**, 592-597 (2019).

5 Wang, H. *et al.* Early transformation of the Chinese power sector to avoid additional coal lock-in. *Environmental Research Letters* **15**, 024007 (2020).

6 Jiang, S., Chen, Z., Shan, L., Chen, X. & Wang, H. Committed CO_2 emissions of China's coal-fired power generators from 1993 to 2013. *Energy Policy* **104**, 295-302 (2017).

7 Zhang, J., Li, X. & Pan, L. Policy effect on clean coal-fired power development in China. *Energies* **15**, 897 (2022).

8 Zhang, S. & Chen, W. Y. China's energy transition pathway in a carbon neutral vision. *Engineering* **14**, 64-76 (2022).

9 Zhuo, Z. *et al.* Cost increase in the electricity supply to achieve carbon neutrality in China. *Nature Communications* **13**, 3172 (2022).

10 Yu, S. *et al.* Synthesis report 2022 on China's carbon neutrality: Electrification in China's carbon neutrality pathways. (2022).

11 Zhang, K., Zhang, W., Shi, Q., Zhang, J. & Yuan, J. Coupling effects of cross-region power transmission and disruptive technologies on emission reduction in China. *Resources, Conservation and Recycling* **189**, 106773 (2023).

12 Liu, F. *et al.* High-resolution inventory of technologies, activities, and emissions of coal-fired power plants in China from 1990 to 2010. *Atmos. Chem. Phys.* **15**, 13299-13317 (2015).

13 Tong, D. *et al.* Current emissions and future mitigation pathways of coal-fired power plants in China from 2010 to 2030. *Environmental Science & Technology* **52**, 12905-12914 (2018).

14 Hopke, P. K. *et al.* Changes in the hospitalization and ED visit rates for respiratory diseases associated with source-specific $PM_{2.5}$ in New York State from 2005 to 2016. *Environmental Research* **181**, 108912 (2020).

15 Henneman, L. *et al.* Mortality risk from United States coal electricity generation. *Science* **382**, 941-946 (2023).

16 The World Bank. State and trends of carbon pricing 2022. (The World Bank, Washington, DC, 2022).

17 Slater, H. et al. 2021 China carbon pricing survey report. (China Carbon Forum, Beijing, China, 2021).
